# Balanced Nuclear and Cytoplasmic Activities of EDS1 Are Required for a Complete Plant Innate Immune Response

**DOI:** 10.1371/journal.ppat.1000970

**Published:** 2010-07-01

**Authors:** Ana V. García, Servane Blanvillain-Baufumé, Robin P. Huibers, Marcel Wiermer, Guangyong Li, Enrico Gobbato, Steffen Rietz, Jane E. Parker

**Affiliations:** Department of Plant-Microbe Interactions, Max-Planck Institute for Plant Breeding Research, Cologne, Germany; The University of North Carolina at Chapel Hill, United States of America

## Abstract

An important layer of plant innate immunity to host-adapted pathogens is conferred by intracellular nucleotide-binding/oligomerization domain-leucine rich repeat (NB-LRR) receptors recognizing specific microbial effectors. Signaling from activated receptors of the TIR (Toll/Interleukin-1 Receptor)-NB-LRR class converges on the nucleo-cytoplasmic immune regulator EDS1 (Enhanced Disease Susceptibility1). In this report we show that a receptor-stimulated increase in accumulation of nuclear EDS1 precedes or coincides with the EDS1-dependent induction and repression of defense-related genes. EDS1 is capable of nuclear transport receptor-mediated shuttling between the cytoplasm and nucleus. By enhancing EDS1 export from inside nuclei (through attachment of an additional nuclear export sequence (NES)) or conditionally releasing EDS1 to the nucleus (by fusion to a glucocorticoid receptor (GR)) in transgenic *Arabidopsis* we establish that the EDS1 nuclear pool is essential for resistance to biotrophic and hemi-biotrophic pathogens and for transcriptional reprogramming. Evidence points to post-transcriptional processes regulating receptor-triggered accumulation of EDS1 in nuclei. Changes in nuclear EDS1 levels become equilibrated with the cytoplasmic EDS1 pool and cytoplasmic EDS1 is needed for complete resistance and restriction of host cell death at infection sites. We propose that coordinated nuclear and cytoplasmic activities of EDS1 enable the plant to mount an appropriately balanced immune response to pathogen attack.

## Author Summary

Plants have evolved a multilayered innate immune system to recognize and respond to potentially destructive microbes in the environment. Resistance to invasive biotrophic and hemi-biotrophic pathogens often involves transcriptional mobilization of defenses and programmed death of host cells at infection sites. However, these processes disturb normal metabolism and growth and therefore have to be tightly controlled. In this study, we examine resistance signaling events inside *Arabidopsis* cells after pathogen activation of intracellular immune receptors. We show that the nucleo-cytoplasmic protein EDS1 acts as an important regulator of transcriptional reprogramming in the immune response by allowing the induction and repression of particular defense-related genes. We provide evidence that EDS1 accomplishes its role as a defense signaling ‘hub’ through coordinated activities in the cytoplasm and nucleus. Maintaining a balance between these two EDS1 pools is probably important for resistance and cell death to a range of infectious microbes and to not ‘overshoot’ defense activation which would be detrimental for the plant.

## Introduction

In animals and plants, innate immune responses of individual cells constitute a major barrier to pathogen infection. Recognition of microbe- or damage-associated molecules is mediated by germ line encoded receptors whose activation is transduced by intracellular signaling systems to an anti-microbial response. Plant innate immunity is expressed as several layers [1]. Membrane pattern recognition receptors (PRRs) with external ligand recognition domains and intracellular kinase domains detect conserved pathogen molecules (Microbe Associated Molecular Patterns or MAMPs) in a similar manner to non-self recognition in animals [2]. PRR activation triggers a resistance response known as MAMP-triggered immunity (MTI) that is normally sufficient to resist colonization by non-adapted microbes. Successful pathogens have evolved effectors that dampen MTI and raise thresholds for activation of defense thereby allowing host invasion [1], [2]. During infection, many pathogen effectors are delivered inside host cells and plants possess intracellular recognition systems mediated by nucleotide-binding and oligomerisation domain (NB or NOD)-leucine rich repeat (LRR) immune receptors [1]. Structurally related NOD-LRR proteins, known also as CATERPILLER, NACHT-LRR or NOD-like receptors (NLRs), serve as pathogen and damage sensors in innate immune responses and cell death control in mammals [3]. Plant *NB-LRR* genes are often located within polymorphic gene clusters [4]. Consistent with a high genetic diversity, particular plant NB-LRRs recognize specific pathogen effectors or their actions on host molecular targets in a process known as effector-triggered immunity (ETI) [1]. The activation of NB-LRRs potentiates host defenses, accelerates defense-associated transcriptional reprogramming and often leads to programmed cell death at attempted infection sites as part of a hypersensitive response (HR) [5], [6], [7]. NB-LRR proteins and host cell death are necessarily under strict control. The biochemical mechanisms underlying receptor activation reveal that NB-LRR proteins behave as molecular switches which are structurally constrained in their inactive forms and activated by release from repression [3], [8]. However, little is known about events between NB-LRR activation and defense induction or the mechanisms which limit resistance signaling to prevent auto-immune reactions.

Recent studies of several plant NB-LRR receptors reveal that they partially localize to and function inside nuclei to trigger innate immune responses [9], [10], [11]. A powdery mildew effector-triggered interaction was observed between barley MLA10 receptor and members of the WRKY family of plant transcription factors [11]. Also, the tobacco N receptor recognizing tobacco mosaic virus (TMV) interacts with certain plant Squamosa Promoter-like (SPL) transcription factors [12], suggesting a close association between some NB-LRRs and the transcription machinery. N resistance requires a host chloroplastic sulfurtransferase that becomes partially relocalized to the cytosol and nucleus by the TMV p50 effector [13]. Thus, dynamic signaling between the cytoplasm and nucleus is likely to be important for innate immune responses [14]. In line with this, *Arabidopsis* plants carrying mutations in genes encoding components of the nucleocytoplasmic trafficking machinery such as the nucleoporins MOS3 (Modifier of *snc1,3*)/SAR3/Nup96 and MOS7/Nup88, and importins MOS6/AtImpα3 and AtImpα4, display defects in resistance to pathogens [15], [16], [17], [18].

The plant immune regulator EDS1 (Enhanced Disease Susceptibility1) is an essential component of basal resistance to virulent (host-adapted) biotrophic and hemi-biotrophic pathogens [19], [20], [21], forming complexes in the cytoplasm and nucleus with its defense co-regulators PAD4 (Phytoalexin Deficient4) and SAG101 (Senescence Associated Gene101) [20], [22]. EDS1 is also required for resistance conditioned by NB-LRRs that possess an N-terminal Toll/Interleukin-1 Receptor (TIR) domain (known as TIR-NB-LRR receptors) [20], [22], [23], [24]. While *Arabidopsis* TIR-NB-LRR receptor RPS4 functions inside nuclei, EDS1 is not necessary for its steady state accumulation or nuclear accessibility [10]. Instead, EDS1 was found to signal after TIR-NB-LRR immune receptor activation and upstream of the transcriptional reprogramming of defense genes, production of resistance hormone salicylic acid (SA) and host cell death [10], [20]. These data are consistent with EDS1 transducing signals generated by activated immune receptors to trigger defense and cell death programs.

Convergence of numerous TIR-NB-LRR receptor activities on EDS1 raises the question of how diverse stimuli are coordinated inside cells to produce a measured immune response. To address this, we have examined where in the cell EDS1 signals in *Arabidopsis* TIR-NB-LRR-conditioned resistance and cell death. We report that there is an increase in the EDS1 nuclear pool during RPS4-triggered resistance to avirulent *Pseudomonas syringae* bacteria which precedes or coincides with EDS1-dependent transcriptional induction and repression of host genes. We also provide evidence for an essential role of nuclear EDS1 in basal and TIR-NB-LRR-conditioned immunity and in reprogramming defense gene expression. While nuclear EDS1 directs transcriptional changes, our data highlight the need also for cytoplasmic EDS1 to induce a complete immune response.

## Results

### Triggering of TIR-NB-LRR resistance to bacteria leads to an early rise in EDS1 nuclear accumulation

**Figure 1:**
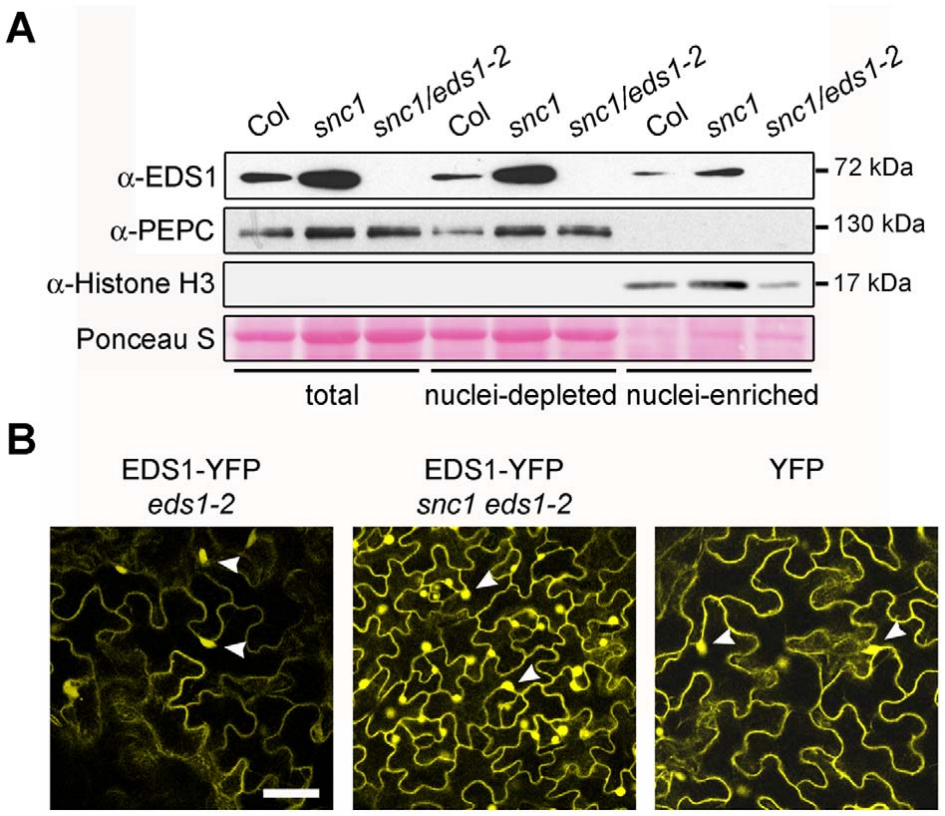
Deregulated resistance in *snc1* leads to increased accumulation of EDS1 in the nucleus and cytoplasm of *Arabidopsis* leaves. (**A**) Western blot showing EDS1 protein levels in total, nuclei-depleted and nuclei-enriched fractions prepared from 4-week-old soil grown plants. PEPC was used as cytosolic marker and Histone H3 as nuclear marker. Molecular weight of protein markers are shown on the right. Nuclei-enriched fractions are 30× concentrated (v/v) compared to nuclei-depleted fractions. (**B**) Confocal images of leaves from *snc1* and *snc1*/*eds1-2* transgenic plants expressing EDS1-YFP fusion under the control of the EDS1 native promoter. A confocal image from a stable transgenic line expressing free YFP protein from 35S promoter is provided for comparison. Images were taken at identical microscope settings. Scale bar is 40 µm and white arrowheads depict nuclei.

The *Arabidopsis snc1* (*suppressor of npr1-1*, *constitutive1*) mutant displays *EDS1*-dependent constitutive resistance and dwarfism due to auto-activation of a TIR-NB-LRR protein [25], [26]. We assessed whether this deregulated immune response is associated with a change in relative amounts of cytoplasmic and nuclear EDS1 that might reflect EDS1 activity in one compartment. As expected, combining Col *eds1-2* [5] with *snc1* (in accession Col-0) to produce a *snc1*/*eds1-2* double mutant led to full suppression of *snc1* dwarfism (Figure S1A) and resistance (data not shown). Western blot analysis of EDS1 protein revealed that total EDS1 amounts were higher in *snc1* compared to wild type (wt) (Figure 1A). However, a proportionate increase in EDS1 accumulation was observed in both nuclei-depleted and nuclear-enriched *snc1* fractions. A stable *eds1-2* transgenic line expressing EDS1 driven by its native promoter and fused at the C-terminus to yellow fluorescent protein (YFP) was selected (EDS1-YFP). It complemented the *eds1* defect in basal resistance (Figure S1E) and was detectable by fluorescence in the cytoplasm and nucleus using a confocal laser-scanning microscope (Figure 1B). Fractionation of EDS1-YFP leaf tissues showed integrity of the EDS1-YFP fusion protein and a similar nucleo-cytoplasmic distribution on Western blots of the same samples probed with anti-EDS1 or anti-GFP antibodies (Figure S1B). Since the native EDS1 (Figure 1A) and EDS1-YFP proteins displayed a similar distribution, we reasoned that EDS1-YFP fluorescence imaging could be used as a reliable indicator of EDS1 localization in leaf cells. The EDS1-YFP line was crossed into *snc1*/*eds1-2* and the subcellular localization of EDS1-YFP protein examined *in vivo* by confocal imaging. Consistent with the distribution of native EDS1 protein (Figure 1A), EDS1-YFP fluorescence was higher in both the cytoplasm and nuclei of *snc1*/*eds1-2* leaf cells compared to *eds1-2* (Figure 1B). These results show that constitutive TIR-NB-LRR resistance increases accumulation of EDS1 protein in both cell compartments. We tested whether the constitutive resistance of *snc1* plants could be explained by increased levels of EDS1. As in *snc1*, the EDS1-YFP line (Figure 1B) and a selected Col *eds1-2* line expressing a functional EDS1-HA fusion under control of the constitutive CaMV 35S promoter, accumulated higher amounts of EDS1 in both nuclei-depleted and nuclei-enriched fractions compared to wt (Figure S1C). However, neither of the two lines displayed dwarfism (Figure S1D) or enhanced basal resistance to virulent *Pseudomonas syringae* pv *tomato* strain DC3000 (*Pst* DC3000) bacteria (Figure S1E). Therefore, raising EDS1 steady state levels does not *per se* produce an auto-immune phenotype. We concluded that the constitutive resistance of *snc1* depends on additional signals generated by the activated TIR-NB-LRR protein.

**Figure 2:**
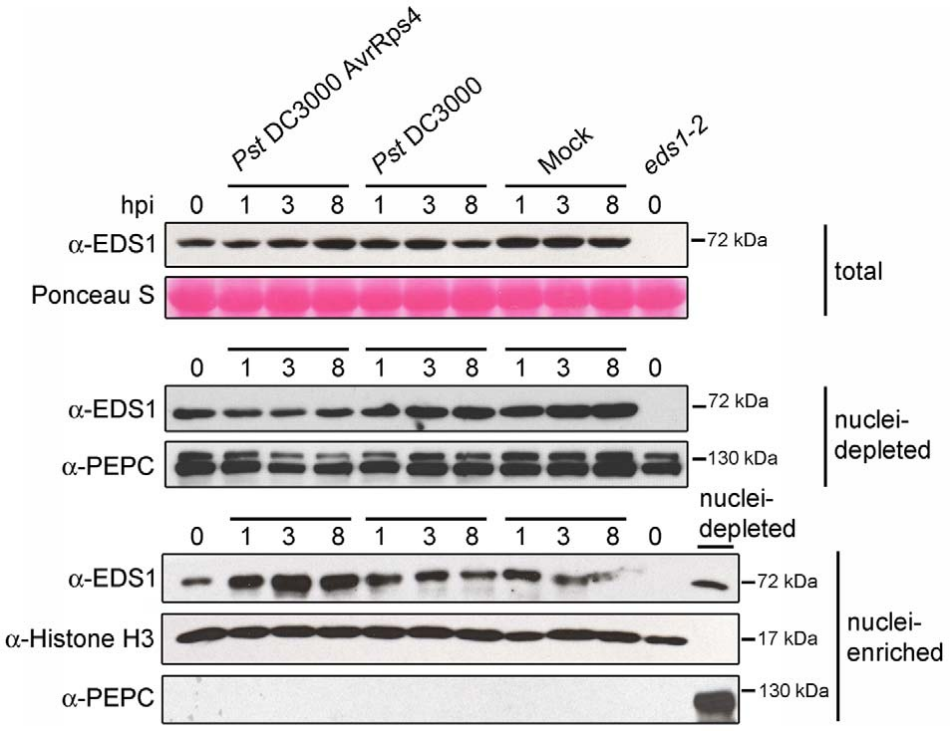
EDS1 nucleo-cytoplasmic distribution after inoculation with virulent and avirulent *Pst* DC3000 bacteria. Western blot showing EDS1 protein levels in total, nuclei-depleted and nuclei-enriched fractions prepared from 4-week-old soil grown plants. Samples were collected at 0, 1, 3 and 8 h post inoculation (hpi) with bacterial suspensions or 10 mM MgCl_2_ (Mock). Ponceau staining of the membrane with total protein extracts shows equal loading. PEPC and Histone H3 were used as cytosolic and nuclear markers, respectively. Nuclei-enriched fractions are 30× concentrated compared to nuclei-depleted fractions. The nuclei-depleted fraction from wt (Col-0) untreated plants was loaded together with nuclei-enriched fractions to check for potential cytosolic contamination with anti-PEPC. Molecular weights of protein markers are shown on the right.

We examined whether there is a change in EDS1 subcellular distribution at an early time point after triggering TIR-NB-LRR resistance that may be short-lived or masked by constitutive activation of the immune pathway. Leaves of wt plants were spray-inoculated with 10 mM MgCl_2_ (mock treatment), virulent *Pst* DC3000 or avirulent *Pst* DC3000 expressing the effector AvrRps4 (*Pst* DC3000 AvrRps4) recognized by TIR-NB-LRR receptor RPS4 [27]. As expected, *Pst* DC3000 AvrRps4 infection produced leaf-spot disease symptoms on *eds1-2* but not on wt leaves after 2–3 days. Total protein, nuclei-enriched and nuclei-depleted fractions were prepared at 0, 1, 3 and 8 h after inoculation. Western blot analysis revealed an increase in EDS1 nuclear amounts 3 h and 8 h after inoculation with *Pst* DC3000 AvrRps4 that was not reflected in changes of EDS1 levels in total or nuclei-depleted fractions (Figure 2). No nuclear enrichment of EDS1 was observed at these time points in response to virulent *Pst* DC3000 or mock treatment (Figure 2). We concluded that an early and potentially important host response to avirulent bacteria involves a change in EDS1 leading to its increase in the nuclear compartment.

### EDS1 nuclear accumulation precedes EDS1-dependent transcriptional reprogramming

**Figure 3:**
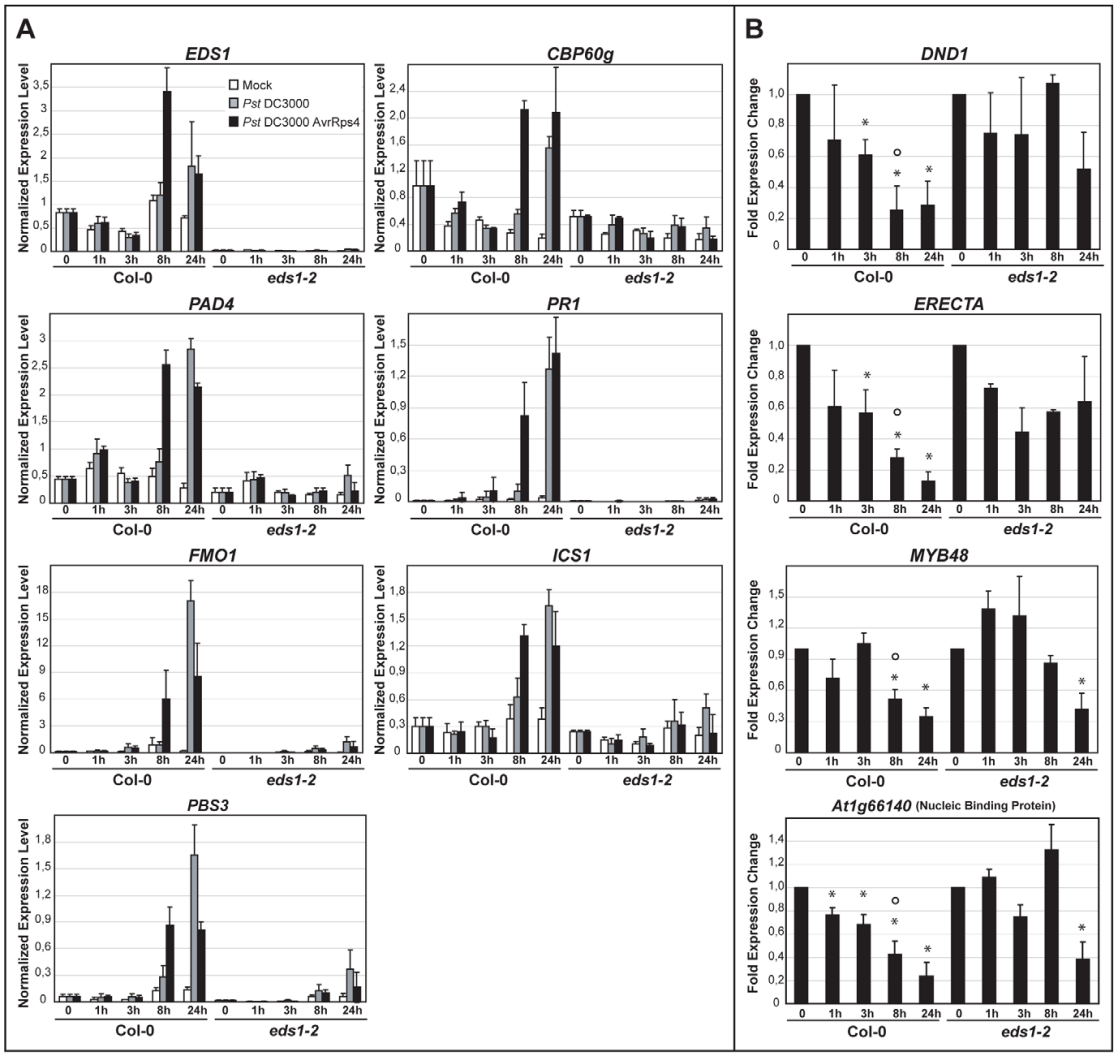
Quantitative transcript profiling of EDS1-dependent genes in response to virulent and avirulent *Pst* DC3000 bacteria. Four-week-old plants were spray-inoculated with 10 mM MgCl_2_ (Mock, white bars), *Pst* DC3000 (grey bars) or *Pst* DC3000 AvrRps4 (black bars) and leaf samples collected at 0, 1, 3, 8 and 24 h post inoculation. Bars represent means and standard deviation from three biological replicates. Transcript levels were determined by qRT-PCR and normalized using the internal control *UBIQUITIN*. (**A**) Relative transcript levels of genes displaying EDS1-dependent induction after infection with either bacterial strain. (**B**) Relative transcript levels of genes displaying EDS1-dependent repression during RPS4-mediated resistance with bars representing the fold change in *Pst* DC3000 AvrRps4-treated samples compared to mock-treated samples. * indicates significant differences between expression at the indicated h post inoculation and 0 h in each genotype and ° indicates significant differences between Col-0 and *eds1-2* at the indicated time point (t-test, p-value <0.01). Slower transcriptional changes in response to *Pst* DC3000 AvrRps4 compared with previous analysis (Table S2, [5]) are probably due to the use of spray inoculation instead of leaf infiltration for bacterial challenge.

Transcriptional profiles of wt and *eds1* responses to *Pst* DC3000 AvrRps4 bacteria infiltrated into leaves [5] were analyzed and candidate genes selected whose expression (at 6 h after infection) was induced or repressed in an *EDS1*-dependent manner (Tables S1 and S2). Prominent among the *EDS1*-dependent upregulated genes are components of SA biosynthesis and signaling (*ICS1*, *PBS3* and *CBP60g*) [28], [29], [30] and *FMO1* which positively regulates an SA-independent branch of EDS1 defense [5], [31]. *PR1*, a commonly used SA response marker [32], was also identified in this group. Among the genes showing *EDS1*-dependent repression was *DND1*, a negative component of plant innate immunity encoding a cyclic nucleotide-gated channel [33] and *ERECTA*, a receptor-like kinase required for resistance to the bacteria *Ralstonia solanacearum* and necrotrophic fungi [34], [35]. A transcription factor (*MYB48*) and predicted nucleic acid binding protein gene (At1g66140), both with unknown functions, were also selected for analysis. In order to validate the expression trends and measure transcriptional changes in relation to EDS1 nuclear accumulation, we quantified transcript levels of the chosen *EDS1*-dependent up- and down-regulated genes in the same leaf tissue extracts that were used for analysis of EDS1 protein (Figure 2). The genes displayed significant *EDS1*-dependent induction (Figure 3A) or repression (Figure 3B) at 8 hpi with *Pst* DC3000 AvrRps4. Induction of the *EDS1*-dependent genes in response to virulent *Pst* DC3000 was not observed at 8 hpi but was seen at 24 hpi, consistent with *Pst* DC3000 triggering a slower transcriptional response [6]. We concluded from these data that *Pst* DC3000 AvrRps4-triggered EDS1 nuclear accumulation precedes or coincides with EDS1-dependent transcriptional reprogramming of defense-related genes. An *EDS1*-dependent increase in *PAD4* transcripts and *EDS1* induction also occurred at 8 hpi with *Pst* DC3000 AvrRps4 (Figure 3A). Thus, EDS1 nuclear enrichment observed 3 h after pathogen challenge (Figure 2) is unlikely to be due to increased *EDS1* gene expression but rather to a post-transcriptional mechanism.

### Nuclear EDS1 is shuttled to the cytoplasm via the CRM1/XPO1 export machinery

**Figure 4:**
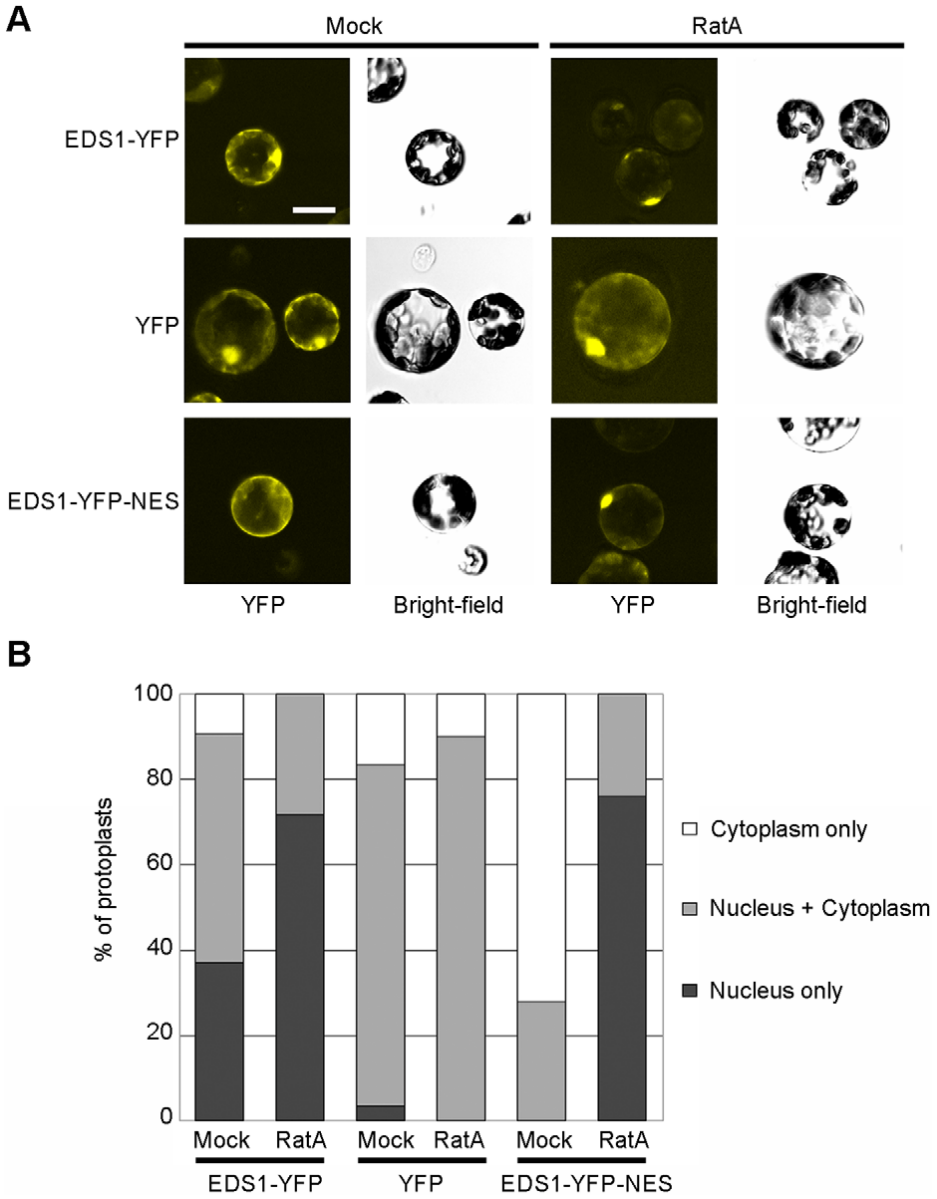
EDS1-YFP shuttles from the nucleus to the cytoplasm in *Arabidopsis* protoplasts. (**A**) YFP fluorescence and bright-field images of *Arabidopsis* protoplasts from stable transgenic lines, as indicated, 4 h after treatment with 15 ng/ml of Ratjadone A (RatA) or methanol (Mock). Bar is 10 µm. (**B**) Percentage of protoplasts showing fluorescence only in the nucleus, only in the cytoplasm or in both compartments determined by counting at least 50 protoplasts per treatment. The experiment was performed three times with similar results.

We reasoned that EDS1 may need to attain a certain concentration in nuclei to fulfill its defense signaling function downstream of TIR-NB-LRR receptor activation. This is supported by the higher nuclear EDS1 amounts observed in *snc1* immune-activated tissues (Figure 1) and lower levels of nuclear EDS1 in an *Arabidopsis mos7* mutant which has a defective Nucleoporin 88 and displays compromised immune responses [16]. In animals, Nup88 modulates CRM1-mediated nuclear export of proteins containing a leucine-rich-type nuclear export sequence (NES) [36]. Thus, EDS1 might possess a functional leucine-rich-type NES and be exported from the nucleus via the *Arabidopsis* CRM1 homolog XPO1 [37] as a mechanism to control nuclear accumulation. The EDS1 amino acid sequence contains two predicted bipartite nuclear localization signals (NLS) at positions 366 and 440 [19] and a putative leucine-rich nuclear export sequence (NES) around amino acid 530 (Figure S2A) that might enable nucleo-cytoplasmic movement. However, mutation of core residues in the EDS1 NLS or NES (Figure S2B) did not lead to obvious relocalization of YFP-tagged EDS1 protein in transient plant expression assays (data not shown). The functionality of an NES can also be determined by assessing protein localization after treatment with the nuclear export inhibitor Ratjadone A (RatA) which inhibits plant and animal XPO1/CRM1 exportins [37], [38]. We examined mesophyll protoplasts generated from the EDS1-YFP line for EDS1-YFP nuclear and cytoplasmic accumulation in the presence or absence of RatA. There was a marked shift in EDS1-YFP fluorescence to nuclei in RatA-treated compared to mock-treated protoplasts (Figure 4), consistent with EDS1 normally being shuttled out of the nucleus via NES-driven nuclear export. As a control, protoplasts generated from a transgenic line expressing YFP under the CaMV 35S promoter did not respond to RatA treatment (Figure 4) because this protein is able to diffuse between the cytoplasm and nucleus [9].

### Enhanced EDS1 export from inside nuclei compromises resistance

**Figure 5:**
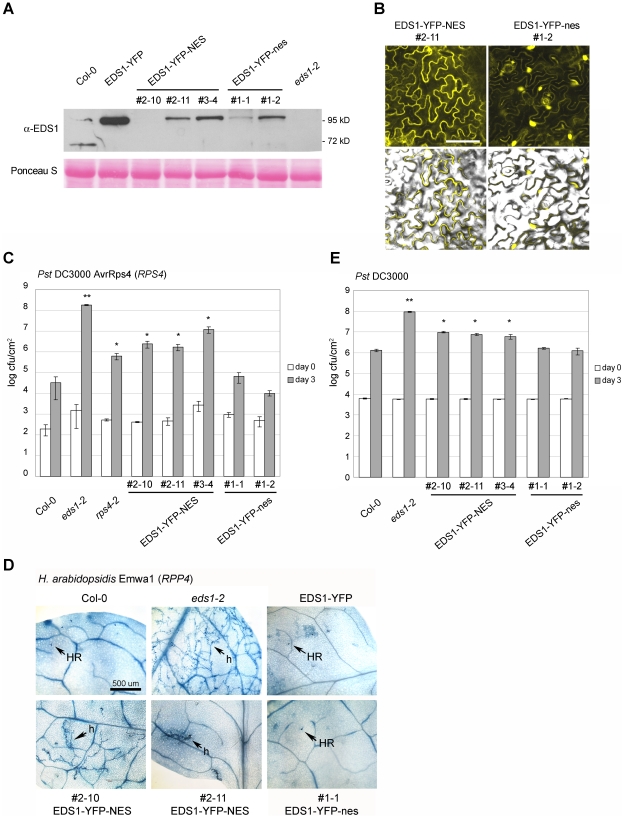
Enhanced export of EDS1 from nuclei leads to reduced resistance. (**A**) Western blot showing total EDS1 protein levels in wt plants and in selected independent transgenic lines expressing the indicated EDS1 fusions. NES and nes denote functional and non-functional nuclear export signals, respectively. Ponceau S staining of the membrane shows equal loading. (**B**) Confocal images showing the subcellular distribution of the EDS1-YFP-NES and EDS1-YFP-nes fusions in one representative transgenic line 5 d post inoculation with virulent *H. arabidopsidis* Noco2. Bar is 15 µm. (**C**) The indicated genotypes were spray-inoculated with avirulent *Pst* DC3000 AvrRps4 bacterial suspension and bacterial titers determined 3 h (day 0, white bars) and 3 d (grey bars) post inoculation. Bars represent means of 5 replicates ± standard error. ** p value<0.001; * p value<0.05. (**D**) Infection phenotypes of leaves inoculated with a spore suspension of avirulent *H. arabidopsidis* isolate Emwa1. Leaves were stained with lactophenol trypan blue 6 d post inoculation to visualize pathogen growth and host cell death. h: hyphae; HR: hypersensitive response. Bar is 500 µm. (**E**) The indicated genotypes were spray- inoculated with a virulent *Pst* DC3000 suspension and bacterial titers determined 3 h (day 0, white bars) and 3 d (grey bars) post inoculation. Bars represent means of 4 replicates ± standard error. ** p value<0.001; * p value<0.05. Similar results were obtained in at least three independent experiments.

To establish whether EDS1 operates in the nucleus or cytoplasm or both compartments in transducing resistance signals, we attempted first to reduce EDS1 nuclear accumulation by increasing the rate of protein nuclear export through fusion of an additional functional NES sequence [11], [39]. An NES (LALKLAGLDI) or mutated ‘nes’ (LALKAAGADA) [39] was attached to the C-terminus of EDS1-YFP and this fusion protein expressed stably in Col *eds1-2* under the control of the EDS1 native promoter. Multiple independent transgenic lines were selected that expressed the EDS1-YFP-NES/nes fusions at levels similar to EDS1 in wt, as monitored on a Western blot (Figure 5A). EDS1-YFP-NES fluorescence was detected in the cytoplasm and in a low proportion of nuclei whereas EDS1-YFP-nes accumulated in both compartments of leaf epidermal cells (Figure 5B and S3A). Protoplasts derived from an EDS1-YFP-NES line exhibited fluorescence in the cytoplasm and at the nuclear rim (Figure 4) consistent with the NES fusion increasing the rate of EDS1 export from nuclei. There was a strong increase in EDS1-YFP-NES nuclear accumulation in this line after RatA treatment (Figure 4), indicating that the NES-tagged protein has the capacity to enter nuclei and that RatA treatment inhibits NES-driven nuclear export. We monitored the distribution of EDS1-YFP-NES (in lines #2–10 and #2–11) and EDS1-YFP-nes (line #1–2) compared to EDS1-YFP in nuclei-depleted and nuclei-enriched fractions on a Western blot probed with anti-EDS1 antibody. This did not reveal obvious differences in nuclear accumulation between the EDS1-YFP-NES and EDS1-YFP-nes extracts (Figure S3B), contrasting with the distinct *in vivo* EDS1-YFP-NES/nes fluorescence patterns (Figure 5B). To investigate reasons for this discrepancy, we imaged fluorescence in sections through individual nuclei in EDS1-YFP-NES line #2–11 and EDS1-YFP-nes line #1–2 on a confocal microscope and compared with the EDS1-YFP line. EDS1-YFP-NES protein fluorescence was detected mostly in the cytoplasm, at the nuclear rim (as seen before in the protoplasts; Figure 4) and inside nuclei in ∼5% of epidermal cells (Figure S3C). EDS1-YFP-nes and EDS1-YFP fluorescence was observed inside the nuclear compartment in most imaged cells (Figure S3C). A similar distribution was found in *eds1-2* epidermal cells transiently expressing EDS1-YFP-NES/nes constructs after particle bombardment (Figure S3D). Together, the data suggest that addition of a functional NES to EDS1-YFP reduces its accumulation inside nuclei but the NES does not allow complete release of EDS1-YFP from the nuclear envelope and associated structures. This may account for its fractionation with nuclei during biochemical separation.

We examined whether resistance mediated by the *TIR-NB-LRR* genes *RPS4* [27] or *RPP4* [40] is affected by increased removal of EDS1 from inside nuclei. The EDS1-YFP-NES lines displayed reduced *RPS4* resistance to *Pst* DC3000 AvrRps4, measured by bacterial growth (Figure 5C), and *RPP4* resistance to *H. arabidopsidis* isolate Emwa1, monitored by trypan blue staining of leaves for pathogen structures and plant cell death (Figure 5D). By contrast, the EDS1-YFP-nes lines were fully resistant (Figure 5C and D). Basal resistance to virulent *Pst* DC3000 (Figure 5E) and *H. arabidopsidis* Noco2 (data not shown) was also compromised in the EDS1-YFP-NES transgenics but was unaffected in EDS1-YFP-nes lines. These data suggest that EDS1 needs to accumulate to a sufficient level inside nuclei to signal a full innate immune response against virulent and avirulent pathogens. With both pathogens, a substantial degree of resistance remained in the EDS1-YFP-NES lines compared to the complete susceptibility of *eds1* mutants.

### EDS1 release to the nucleus drives *TIR-NB-LRR* and basal resistance responses

We reasoned that the residual pathogen resistance in the EDS1-YFP-NES lines (Figure 5) might be conferred by a low amount of EDS1-YFP-NES protein initially entering nuclei before it is exported or partially trapped at the nuclear envelope (Figure 4 and S3), arguing for an entirely nuclear function of EDS1. Alternatively, the intermediate resistance could reflect a contribution of cytoplasmic EDS1 to the immune response. We therefore used a different strategy to control EDS1 localization inside cells by fusing the C-terminus of EDS1-YFP or YFP alone (as control) to the steroid binding domain of the mammalian glucocorticoid receptor (GR) in a cassette driven by the CaMV 35S promoter [41]. Proteins fused to GR are normally retained in the cytoplasm through association with an Hsp90 chaperone complex [42] and treatment of plant cells with the steroid hormone Dexamethasone (Dex) drives nuclear localization of the GR fusion protein [41], [43].

**Figure 6:**
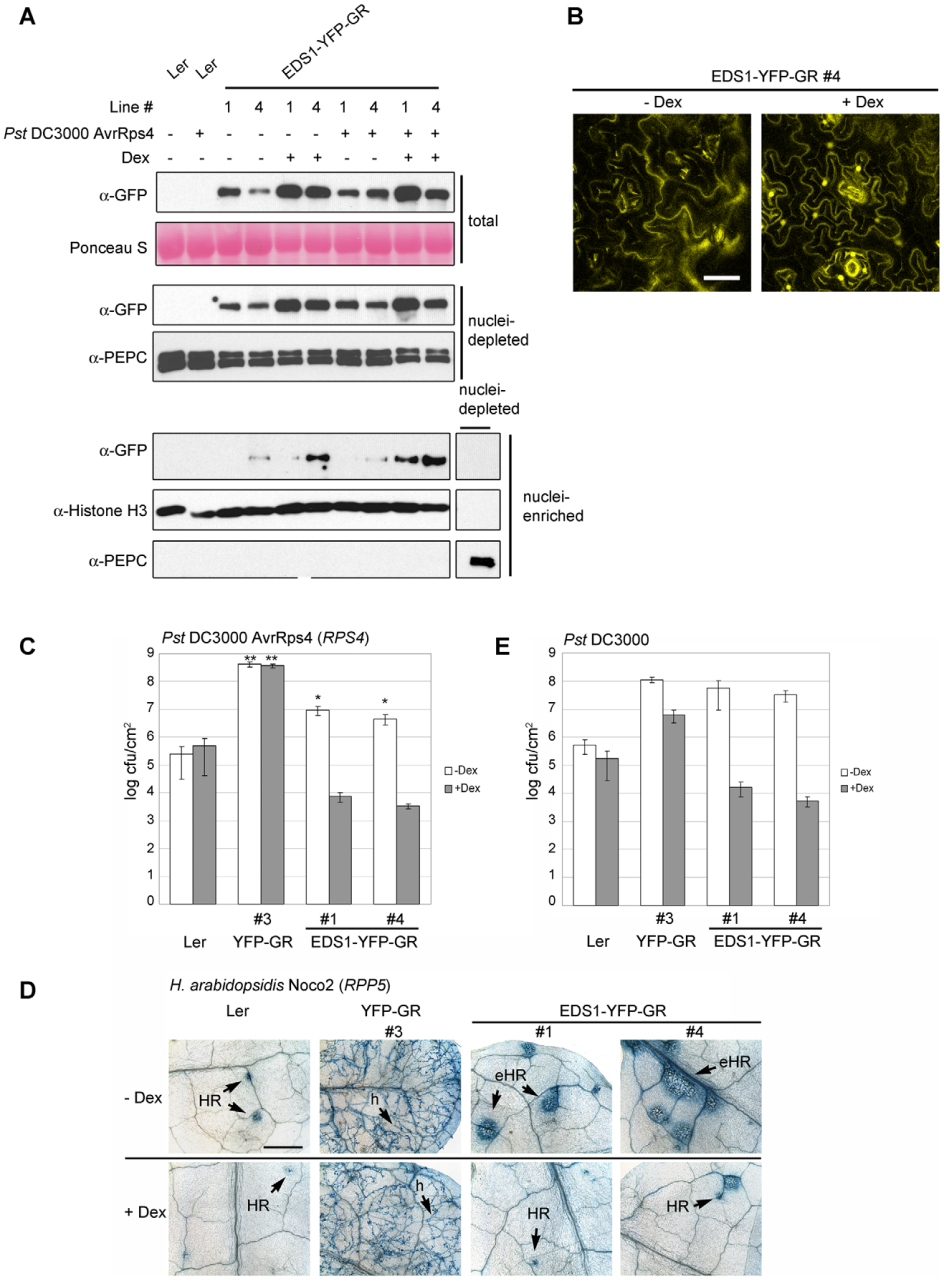
Nuclear release of EDS1-YFP-GR by Dexamethasone (Dex) treatment leads to protein accumulation in nuclei and enhanced disease resistance. (**A**) Leaves of 4-week-old plants were pre-treated with Dex (+) or mock solution (−) and spray-inoculated with buffer (−) or *Pst* DC3000 AvrRps4 (+) and protein samples prepared 8 h post inoculation, as indicated. Western blot shows EDS1 protein levels in total, nuclei-depleted and nuclei-enriched fractions in transgenic lines #1 and #4 expressing the EDS1-YFP-GR fusion protein. Ponceau S staining of the membrane shows equal loading. PEPC and Histone H3 signals were used, respectively, as cytosolic and nuclear marker. (**B**) Confocal images showing EDS1-YFP-GR subcellular distribution in a representative transgenic line with or without Dex treatment 13 h after Dex treatment. Bar is 30 µm. (**C**) Untreated (white bars) or Dex-pretreated (5 h, grey bars) 4-week-old plants of the indicated genotypes were spray-inoculated with avirulent *Pst* DC3000 AvrRps4 and bacterial titers determined 4 d post inoculation. Bars represent means of 4 replicates ± standard error. ** p value <0.001; * p value <0.05. (**D**) Untreated or Dex-pretreated (5 h) 2-week-old plants were spray-inoculated with a spore suspension of avirulent *H. arabidopsidis* isolate Noco2. Leaves were stained with lactophenol trypan blue 7 d post inoculation to visualize pathogen growth and host cell death. h: hyphae; HR: hypersensitive response; eHR: expanded HR. Bar is 500 µm. (**E**) Untreated (white bars) or Dex-pretreated (5 h, grey bars) 4-week-old plants were spray-inoculated with virulent *Pst* DC3000 bacterial suspension and bacterial titers determined 4 d post inoculation. Bars represent means of 4 replicates ± standard error. ** p value <0.001; * p value <0.05. Results of the bacterial and oomycete infection assays are representative of three independent experiments.

From multiple independent transgenic lines expressing EDS1-YFP-GR or YFP-GR in an *eds1-2* background (in *Arabidopsis* accession L*er*) two lines (#1 and #4) were selected that had detectable EDS1-YFP-GR protein on a Western blot of leaf extracts probed with anti-GFP antibody (Figure 6A). Extracts were also probed with anti-EDS1 antibody which gave weaker signals than with anti-GFP and showed that EDS1-YFP-GR protein accumulated at a rather low level (Figure S4A) despite being driven by the 35S promoter (Figure S4B). A single line expressing YFP-GR (Figure S4C) was taken as control. Before Dex treatment, weak YFP fluorescence was observed in the cytoplasm of the EDS1-YFP-GR lines, as shown for line #4 (Figure 6B). YFP fluorescence was also detected in nuclei at 5 h (not shown) and 13 h (Figure 6B) after spraying leaves with 30 µM Dex. At 13 h (after Dex) this was associated with an increase in EDS1-YFP-GR protein in total, nuclei-depleted and nuclei-enriched fractions (Figure 6A) but not with a change in *EDS1-YFP-GR* transcript levels (Figure S4B). The EDS1-YFP-GR transgenic lines developed normally before or after treatment with the steroid hormone. We therefore used them to investigate the roles of cytoplasmic and nuclear EDS1 in the plant immune response.

EDS1-YFP-GR plants were first analyzed for their response to avirulent *Pst* DC3000 AvrRps4. Leaves were pre-treated with 30 µM Dex for 5 h before bacterial inoculation and bacterial titers were counted at day 4. In the absence of Dex, the EDS1-YFP-GR lines displayed resistance to *Pst* DC3000 AvrRps4 that was intermediate between wt and *eds1-2* (Figure 6C). Treatment with Dex reduced bacterial numbers by ∼1000-fold to levels below those seen in wt (Figure 6C). Wt (L*er*) and control YFP-GR (L*er eds1-2*) backgrounds exhibited, respectively, similar levels of resistance and susceptibility with or without Dex treatment (Figure 6C). Also, Dex application to wt plants did not alter levels of native EDS1 protein (Figure S4D), indicating that the resistance of the EDS1-YFP-GR lines is conditioned by Dex-induced release of EDS1. Resistance mediated by a different *EDS1*-dependent *TIR-NB-LRR* gene (*RPP5*) [44] to *H. arabidopsidis* isolate Noco2 was also conditional on Dex treatment, with pathogen growth being efficiently contained by a plant hypersensitive response at infection sites (HR; Figure 6D). As with *Pst* DC3000 AvrRps4 bacteria, the EDS1-YFP-GR lines displayed partial resistance to *H. arabidopsidis* in the absence of Dex. Notably, this was associated with spreading cell death at infection foci (Figure 6D). Without Dex treatment, basal resistance in these lines to virulent *Pst* DC3000 was suppressed to similar levels as in *eds1-2* and was equivalent to resistance in wt after Dex application (Figure 6E).

The EDS-YFP-GR signal in nuclei-enriched fractions was strongest when Dex treatment was followed by inoculation with *Pst* DC3000 AvrRps4 (Figure 6A and S4A). Since EDS1-YFP-GR is expressed under the control of a constitutive promoter, it is unlikely that the increase is due to transcriptional up-regulation. Indeed, no significant change in expression of the *EDS1-YFP-GR* transgenes was observed in leaves 8 h after challenge with *Pst* DC3000 AvrRps4, with or without Dex pretreatment (Figure S4B). These data suggest that recognition of AvrRps4 or another pathogen stimulus enhances nuclear accumulation of Dex-released EDS1-YFP-GR.

**Figure 7:**
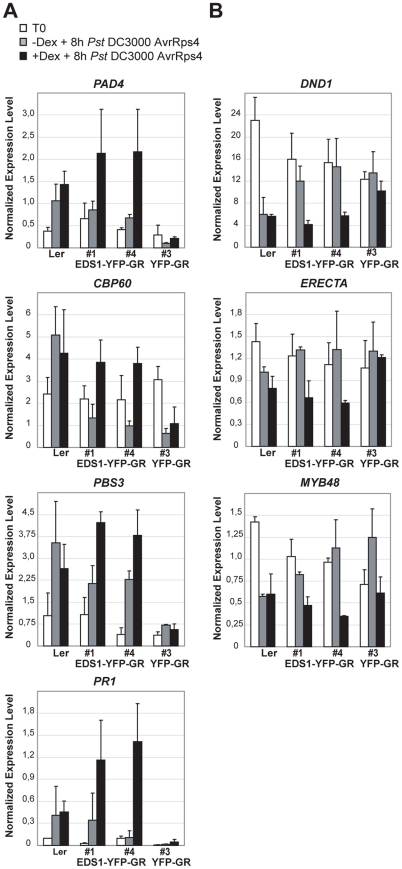
Quantitative transcript profiling of EDS1-dependent genes in EDS1-YFP-GR transgenic lines after triggering RPS4 resistance. Four-week-old plants untreated (grey bars) or pre-treated with Dexamethasone (Dex) for 5 h (black bars) were inoculated with *Pst* DC3000 AvrRps4. Leaf samples were collected from untreated plants (T0) and 8 h after pathogen inoculation. Bars represent means and standard deviation of two or three biological replicates. Relative transcript levels were determined by qRT-PCR and normalized using the internal control *UBIQUITIN*. Expression was normalized in different samples using the endogenous control gene *UBIQUITIN*. (**A**) Up-regulated genes. (**B**) Down-regulated genes.

We monitored expression of genes displaying *EDS1*-dependent transcriptional changes 8 h after *Pst* DC3000 AvrRps4 inoculation (Figure 3) in the EDS1-YFP-GR transgenic lines with or without 5 h Dex pre-treatment. The 13 h time point after Dex application is when EDS1-YFP-GR protein signals were monitored on Western blots and by fluorescence imaging (Figure 6A and B). The results show Dex-dependent reprogramming of EDS1-induced and EDS1-repressed genes in response to DC3000 AvrRps4 (Figure 7). Whereas cytosolic retention of EDS1-YFP-GR substantially reduced pathogen-induced expression changes, Dex-induced nuclear accumulation of EDS1-YFP-GR allowed similar or larger transcriptional changes than in DC3000 AvrRps4-challenged wt plants (Figure 7). We concluded that nuclear EDS1 is needed to drive pathogen-induced reprogramming of transcription.

## Discussion

Plant innate immunity to invasive biotrophic or hemi-biotrophic pathogens involves the rapid mobilization of defense and cell death programs. However, these are energetically costly and disturb normal development and fitness and are therefore under strict genetic control [45], [46]. We provide evidence that *Arabidopsis* EDS1 coordinates immune responses by functioning in both the nucleus and the cytoplasm. Although nuclear EDS1 is required for TIR-NB-LRR-induced reprogramming of defense gene expression and resistance, a cytoplasmic EDS1 pool is maintained during infection and development, and is needed for complete resistance to bacterial and oomycete pathogens.

### Maintaining EDS1 nucleo-cytoplasmic balance

We used a combination of biochemical fractionation of leaf tissue extracts and in vivo imaging of fluorescent-tagged proteins in leaf cells to assess EDS1 accumulation in the cytoplasmic and nuclear compartments. Enhanced EDS1 expression in *snc1* mutants (with constitutive resistance due to an auto-activate TIR-NB-LRR protein (Figure 1)) [26] or reduced EDS1 in *mos7* mutants (with compromised resistance because of a defect in Nup88-mediated nuclear retention [16]) resulted in similar EDS1 nuclear and cytoplasmic ratios to those found in healthy wt plants. We also observed higher EDS1 accumulation in both cell compartments at late stages of infection with virulent *H. arabidopsidis* (data not shown), suggesting that nuclear and cytoplasmic EDS1 pools need to be equilibrated during prolonged activation of defense and development. These EDS1 accumulation patterns, together with our earlier finding that EDS1 forms different complexes with its signaling partners PAD4 and SAG101 in the cytoplasm and nuclei of healthy leaf cells [22], suggest distinct but cooperative roles of EDS1 cytoplasmic and nuclear pools. Analysis of mesophyll protoplasts derived from the EDS1-YFP and EDS1-YFP-NES transgenic lines (Figure 4) showed that EDS1 is capable of nuclear transport receptor (CRM1/XPO1)-mediated shuttling between the cytoplasm and nucleus through the nuclear pores. It is therefore likely that cytoplasmic and nuclear EDS1 pools communicate through the nuclear pores to coordinate resistance and cell death programs. There may be a requirement for optimal cycling of EDS1 between the nuclear and cytoplasmic compartments, consistent with dynamic signaling across the nuclear envelope being critical for animal and plant immune responses [14].

### Nuclear EDS1 directs transcriptional reprogramming

An increase in nuclear EDS1 accumulation either by pathogen infection triggering TIR-NB-LRR activation (Figure 2) or by Dex-induced release of a EDS1-YFP-GR fusion to nuclei accompanying a pathogen stimulus (Figure 6) correlated with the induction or repression of particular host genes (Figures 3 and 7), supporting a role for nuclear EDS1 in driving transcriptional reprogramming during plant defense. Among the EDS1-induced genes are components of SA biosynthesis and signaling (e.g *ICS1*, *PAD4*, *PBS3* and *CBP60g*) [28], [29], [30], [47] (Table S2). Hence, a key step of EDS1 nuclear action is to stimulate the SA pathway. Although SA has a minor role in local TIR-NB-LRR triggered resistance and cell death [20], [40], [48] it is a central component of plant systemic resistance to biotrophic pathogens [49]. Accordingly, EDS1 is required for systemic signaling beyond pathogen infection sites [50], [51]. EDS1-directed repression of genes such as *DND1* [33] and *ERECTA* [34], [35] which have a negative impact on resistance to biotrophic pathogens but contribute to resistance to some necrotrophic pathogens, suggests a ‘master’ role of EDS1 complexes in coordinating gene expression outputs. Control of *ERECTA* expression may be of particular significance since this gene affects plant growth and development as well as responses to environmental stimuli through a network of cis- and trans-regulation [52]. The precise mode of action of EDS1 inside nuclei remains unclear. The primary EDS1 amino acid sequence does not have obvious DNA-binding domains [19] and analysis of nuclear EDS1 has not so far revealed specific association with chromatin (S. Blanvillain-Baufumé, R.P. Huibers and J.E. Parker, unpublished). However, interactions have been found between EDS1 and a number of transcription factors in yeast 2-hybrid assays (S. Blanvillain-Baufumé, R.P. Huibers and J.E. Parker, unpublished), suggesting a mechanism by which EDS1 could modulate transcription. Nuclear EDS1 complexes may work by binding transcription factors and/or repressors in the nucleoplasm to guide activities and associations with the DNA. Restraining proteins away from their site of action has emerged as an important mechanism for controlling transcriptional activators such as NF-KB in mammalian cells [53] and a number of transcriptional regulators in plants (such as bZIP10, WRKY33, NPR1), and emphasizes the extent of intracellular protein dynamics during stress signaling [14]. The role of the EDS1 signaling partners PAD4 and SAG101 in this process is also not clear. They form transient complexes with EDS1 that distribute differently between the cytoplasm and nucleus and, together, are essential for immune response and cell death activation [20], [22]. One model is that PAD4 and SAG101 help EDS1 to circulate between the cytoplasm and nucleus to coordinate the binding and release of transcription factors. It will be important to test whether EDS1 mislocalization affects association with PAD4 or SAG101 and if these components participate in EDS1 interactions with transcription factors in the cell.

Evidence points to EDS1 operating downstream of activated RPS4 [10]. We therefore postulate that a change in EDS1 nucleo-cytoplasmic status leading to transcriptional reprogramming is triggered by RPS4 recognition of AvrRps4. However, we cannot exclude the possibility that an alteration in EDS1 occurs as part of the plant response to bacterial MAMPs. An early MAMP-induced change might be effectively dampened by virulent DC3000 bacteria and reinstated (or amplified) by RPS4 responding to AvrRps4 [1]. Whatever the stimulus, a rise in EDS1 alone seems insufficient to trigger resistance because a mild increase in EDS1 levels (in the pEDS1:EDS1-YFP transgenic line) or strong over expression of EDS1 (in the p35S:EDS1-HA line) resulting in over-accumulation of EDS1 in the nuclear and cytoplasmic compartments did not produce an auto-immune response (Figure S1C–E). These data suggest that R protein activation is a necessary step for EDS1 inside nuclei to reprogram transcription. In this regard, nuclear EDS1 levels appear to be controlled post-transcriptionally. This holds for the early nuclear accumulation of EDS1 after RPS4 activation because it occurs before changes in *EDS1* transcript levels are observed (Figures 2 and 3). It is therefore distinct from pathogen-induced increases in *EDS1* and *PAD4* mRNAs and total protein at later time points which are attributed to a positive feedback loop involving SA to amplify host resistance [5], [20], [47]. Post-transcriptional regulation likely also accounts for the rise in EDS1-YFP-GR steady state levels after its Dex-induced release to the nucleus because bacterial and Dex treatments did not alter *EDS1-YFP-GR* mRNA abundance (Figure S4B). The low level of EDS1-YFP-GR protein accumulating in the cytoplasm before Dex treatment (Figure 6 and S4) may be a consequence of impeding nuclear access or nucleo-cytoplasmic cycling of EDS1. In line with the tendency for nuclear and cytoplasmic EDS1 to equilibrate, we postulate that an initial rise in nuclear EDS1-YFP-GR causes a rapid adjustment of the cytoplasmic EDS1 pool, thereby permitting a complete immune response to be activated (Figure 6). Modification of EDS1 leading to increased protein stabilization likely alters its functions inside nuclei to permit reprogramming of transcription. A post-transcriptional process was also proposed to account for lowered total cellular accumulation of EDS1 and the SA response regulator NPR1 in the *Arabidopsis mos7* (*nup88*) nucleoporin mutant since their corresponding transcript levels were not reduced [16]. A growing body of evidence shows that targeted degradation by the proteasome coordinates the exchange of transcription components on and off the chromatin, allowing dynamic shifts from repression to activation of genes [54], [55]. A recent analysis indeed shows that proteasome-mediated turnover of NPR1 in the nucleus is necessary for its function as a transcriptional co-activator in systemic resistance [56].

### Role for cytoplasmic EDS1 in modulating pathogen-induced cell death

Partial resistance to bacteria and oomycete pathogens exhibited by the EDS1-YFP-NES (Figure 5) and EDS1-YFP-GR lines (in the absence of the Dex release stimulus; Figure 6) points to a role of cytoplasmic EDS1 in promoting an efficient immune response. It is possible that transient EDS1 nuclear pools in the EDS1-YFP-NES lines (Figure 4) or leakiness in EDS1-YFP-GR cytoplasmic retention (though undetectable in the microscope – Figures 6B) could account for the residual resistance. Also, fusion of an additional NES to EDS1 may have unexpected consequences since, although it reduces levels of EDS1-YFP inside nuclei, it appears to impede efficient release of EDS1 from the nuclear rim (Figure 4 and S3). Nevertheless, the fact that EDS1 is actively shuttled from the nucleus to the cytoplasm where it forms different complexes [22] and a cytoplasmic EDS1 pool is maintained throughout infection and development, argue for EDS1 functions in the cytoplasm. A role for cytoplasmic EDS1 is most evident in TIR-NB-LRR resistance to *Pst* DC3000 AvrRps4 (Figures 5C and 6C) and *H. arabidopsidis* (Figures 5D and 6D) but less clear in EDS1-dependent basal resistance (Figures 5E and 6E). The cytoplasmic EDS1 pool may counter-balance activities of nuclear EDS1 by, for example, sequestering transcription factors outside the nuclei. Alternatively, but not exclusively, EDS1 cytosolic complexes might have a unique signaling function needed for a complete immune response. Particularly noticeable was the expansion of cell death lesions at sites of infection by the obligate biotrophic pathogen *H. arabidopsidis* in EDS1-YFP-GR lines in the absence of Dex (Figure 6D). These lesions extended beyond obvious pathogen structures and were therefore different from the characteristic trailing necrotic phenotype observed in *Arabidopsis* mutants with relaxed TIR-NB-LRR resistance [20]. No cell death was observed in the EDS1-YFP-GR lines before pathogen inoculation indicating that the cell death requires a pathogen stimulus. One scenario is that failure to restrict host cell death in these lines is due to diminished EDS1 nuclear function in regulating genes that suppress or contain cell death. Supporting this, a recent study showed that SA antagonism of EDS1-driven cell death initiation is needed for a complete immune response to biotrophic pathogens [57]. It is possible that cytosolic EDS1 actively promotes cell death in response to oxidative stress signals emanating from the chloroplasts [57], [58], [59] and this is counter-balanced by transcriptional reprogramming in the nucleus to moderate potentially destructive cellular events. Future work aims to characterize the cytoplasmic and nuclear activities of EDS1 complexes which seem to be carefully poised in the cell for optimal responsiveness to biotic stress.

## Materials and Methods

### Plant growth and pathology assays

*Arabidopsis* wild type accessions, *eds1-2* [5] and *rps4-2* [10] mutants have been described. Plants were grown in soil in controlled environment chambers under a 10 h light regime (150–200 µE/m^2^s) at 22°C and 65% relative humidity. *Pst* DC3000 and *Pst* DC3000 AvrRps4 strains were grown for 24 h at 28°C on NYGA solid medium supplemented with the corresponding antibiotics. For bacterial growth assays and expression analyses, 4-week-old plants were spray-inoculated with bacterial suspensions at 4×10^8^ cfu/ml in 10 mM MgCl_2_ with 0,04% (v/v) Silwet L-77 (Lehle Seeds) or mock-treated with 10 mM MgCl_2_ containing 0,04% (v/v) Silwet L-77. *In planta* bacterial titers were determined at the indicated time points after inoculation by shaking leaf discs in 10 mM MgCl_2_ with 0,01% (v/v) Silwet L-77 at 28°C for 1 h [60]. At least five plants per genotype were used for each sampling. Bacterial numbers were compared between lines using a two-tailed Student's t-test. *H. arabidopsidis* isolates Emwa1 and Noco2 were maintained and inoculated onto 2-week-old plants at 4×10^4^ spores/ml as described [22]. Plant cell death and *H. arabidopsidis* infection structures were visualized under a light microscope after staining leaves with lactophenol trypan blue [23].

### *Arabidopsis* stable transgenic lines

Binary vectors suitable for Gateway cloning (Invitrogen) and protein localization studies were generated. Monomeric yellow fluorescent protein (YFP) was PCR-amplified from vector pcDNA3-mYFP (obtained from Dr. Irine Prastio, Howard Hughes Medical Institute, UC San Diego, CA) and ligated into the binary vector pXCS-HisHA containing a CaMV 35S promoter [61], generating pXCS-YFP. A Gateway recombination cassette (reading frame B as *Eco*RV-fragment, Invitrogen) was ligated and the CaMV 35S promoter removed, resulting in the Gateway destination vector pXCG-YFP. Genomic Ler EDS1 sequence including 1.4 kb of endogenous promoter and 2.1 kb of coding sequence without stop codon was cloned in pENTR/D-TOPO (Invitrogen) and an LR reaction was performed to generate the vector pXCG-pEDS1-EDS1-YFP. Constructs were used to transform Col *eds1-2* plants [5] using the floral-dip method [62]. Several independent EDS1-YFP transgenic lines were generated that fully complemented the *eds1-2* mutation and a representative line used for further analysis. Functional NES from PKI (LALKLAGLDI) and the non-functional nes (LALKAAGADA) [39] were attached to the C-terminus of mYFP through PCR amplification of vector pcDNA3-mYFP. The same strategy as described above was followed to generate construct pXCG-pEDS1-EDS1-YFP-NES/nes and stable *Arabidopsis* Col *eds1-2* stable transgenic lines. To generate transgenic plants expressing EDS1-HA under the CaMV 35S promoter, an LR reaction was made between the pENTR/D-TOPO (Invitrogen) vector containing genomic Ler EDS1 coding sequence [22] and the pXCS-3xHA vector [61]. Constructs were transferred to *A. tumefaciens* strain GV3101 (pMP90RK) and transformed into *Arabidopsis* Col *eds1-2* plants. To generate transgenic plants expressing StrepII-3xHA-YFP driven by the CaMV 35S promoter, the plasmid pENS-StrepII-3xHA-GW was made using the vector pXCS-3xHA [61]. YFP was PCR-amplified from vector pcDNA3-mYFP and cloned in pENTR/D-TOPO (Invitrogen) and an LR reaction performed to obtain the pENS-StrepII-3xHA-YFP plasmid. Constructs were transformed into *Arabidopsis* Col plants, as described above. GR fusions were generated using the vector pBI-ΔGR [41] and cEDS1-YFP amplified from the pEXG-cEDS1-YFP vector. Constructs were transferred to *A. tumefaciens* strain GV3101 (pMP90) and used to transform L*er eds1-2* plants [19].

### Protein expression analysis

Total protein extracts were prepared by grinding leaf material in liquid nitrogen. Samples were resuspended in equal volumes of 2× Laemmli loading buffer, boiled for 5 min and centrifuged to remove cell debris. Proteins were separated by SDS-PAGE and electroblotted to nitrocellulose membranes for protein gel blot analysis. Equal loading was monitored by staining membranes with Ponceau S (Sigma-Aldrich). Nuclear fractionation of *Arabidopsis* tissue was performed as previously described [22] using 4-week-old plants. Nuclei-enriched fractions were 30× more concentrated than nuclei-depleted fractions based on the final volume of each fraction. Antibodies used for immunoblot analysis were as described: anti-EDS1 [22], anti-PEPC (Rockland; [63]), anti-Histone H3 (Abcam; [22]), anti-PICKLE [64] and anti-CSN4 (BIOMOL International).

### Generation of *Arabidopsis* protoplasts and shuttling assay

*Arabidopsis* leaf mesophyll protoplasts were prepared from 4-week-old plants grown in a normal light/dark regime, according to Asai *et al* [65] with some modifications. Leaf strips were digested in enzyme solution (0.4 M Mannitol; 20 mM KCl; 20 mM MES pH 5.7) with 1.5% cellulase (Onozuka R-10, Merck, Darmstadt, Germany) and 0.4% Macerozyme (R-10, Serva, Heidelberg, Germany). The solution was vacuum infiltrated for 3 min, incubated for 30 min with vacuum pressure and then for 2 h with gentle shaking. The protoplast solution was filtered through a 62 µm nylon mesh and washed with W5 solution (154 mM NaCl, 25 mM CaCl_2_·2H_2_O, 5 mM KCl, 2 mM MES pH 5.7). Isolated protoplasts were resuspended in Mannitol solution (0.4 M Mannitol, 15 mM MgCl_2_·6H_2_O, 4 mM MES, pH 5.8). The nuclear export inhibitor Ratjadone A (Alexis Biochemicals) was dissolved in methanol (10 ng/µl) and added to protoplasts to a final concentration of 15 ng/ml. Control samples were mock treated-with an equal concentration of methanol.

### Confocal imaging

*Arabidopsis* leaves or protoplast solutions were examined with a confocal laser-scanning microscope Leica TCS 4D.

### Gene expression analyses

Total RNA was extracted from plant leaves using TRI-reagent (SIGMA) and RNA was reverse transcribed into cDNA using SuperScriptII (Invitrogen) following the manufacturer's instructions. Quantitative RT-PCR experiments were performed in an iQ5 Real-Time PCR Detection System (Bio-Rad) using Brilliant SYBR Green QPCR Core Reagent (Stratagene) as dye. Experiments were performed using three independent biological samples. Relative transcript levels were calculated using the iQ5 Optical System Software (Version 2.0). *Ubiquitin UBQ10* (At4g05320) transcript levels were used as internal reference. Primers used in these experiments are available on request.

## Supporting Information

Figure S1 Increased EDS1 protein levels do not lead to constitutive resistance. **(A)** Picture of 3-week-old soil grown plants showing that Col *eds1-2* suppresses *snc1* phenotypes and expression of EDS1-YFP (driven by the EDS1 native promoter) in *snc1* restores them. **(B)** Western blot showing levels of EDS1-YFP fusion protein in total, nuclei-depleted and nuclei-enriched fractions prepared from 4-week-old soil grown plants. Nuclei-enriched fractions are 30× concentrated (v/v) compared to nuclei-depleted fractions. Samples were loaded twice on the same gel and blotted together. One half of the membrane was probed with anti-EDS1, the other with anti-GFP antibodies. PEPC and Histone H3 were used respectively as cytosolic and nuclear markers. Molecular weights of protein markers are shown on the right. **(C)** Western blot showing EDS1 protein levels in nuclei-depleted and nuclei-enriched fractions prepared from healthy tissue of the indicated genotypes over expressing EDS1. PEPC was used as cytosolic marker. Chromatin associated Histone H3, the chromatin remodeler protein PKL (PICKLE) and the CSN4 subunit of COP9 signalosome (chromatin non-associated protein) were used as nuclear markers. The nuclei-depleted fraction from wt (Col-0) untreated plants was loaded together with nuclei-enriched fractions in order to monitor potential cytosolic contamination by anti-PEPC signal. Molecular weights of protein markers are shown on the right. **(D)** Picture of 4-week-old soil grown plants of the indicated genotypes showing normal growth of all lines except *snc1*. **(E)** Bacterial infection assay. 4-week-old soil grown plants were spray-inoculated with *Pst* DC3000 AvrRps4 and bacterial titers determined 0 and 3 d post inoculation. Bars represent means of 3 replicates ± standard error. ** p value<0.001.
(3.04 MB TIF)

Figure S2 Domain structure of EDS1 protein depicting putative localization signals. **(A)** Black box represents conserved lipase-like domain, red lines show positions of predicted nuclear localization signals (NLSs) and dashed red box represents predicted nuclear export signal (NES). **(B)** Amino acid sequences of EDS1 putative NLS and NES motifs. Residues in italics were mutagenized to test the functionality of the signal. Lysine (K) and arginine (R) residues in NLS1 and NLS2 were replaced with glutamine and Leucine (L) 530 in NES sequence was replaced with alanine.
(0.03 MB TIF)

Figure S3 Nucleo-cytoplasmic partitioning of the EDS1-YFP-NES and EDS1-YFP-nes fusion proteins. **(A)** Percentage of cells showing detectable nuclear fluorescence in the indicated stable transgenic lines from at least 100 epidermal cells in three individual plants per genotype. **(B)** Western blot showing EDS1 protein levels in nuclei-depleted and nuclei-enriched fractions. PEPC and Histone H3 were used respectively as cytosolic and nuclear markers. Molecular weights of protein markers are shown on the right. **(C)** Confocal images showing the subcellular distribution of the EDS1-YFP-NES and EDS1-YFP-nes fusion proteins in leaves of healthy transgenic plants. Bar is 15 µm. Accumulation of EDS1-YFP-NES fusion protein in and around nuclei can be observed and is depicted with a white arrowhead. **(D)** Confocal images showing the subcellular distribution of the indicated fusion proteins expressed transiently in Col *eds1-2* leaf epidermal cells by particle bombardment.
(1.50 MB TIF)

Figure S4 Accumulation of EDS1 and EDS1-YFP-GR fusion after Dex treatment. **(A)** Identical samples as used in Figure 6A probed with anti-EDS1. Four-week-old plants were pretreated with Dex (5 h) and spray-inoculated with *Pst* DC3000 AvrRps4. Protein samples were prepared 8 h post inoculation with *Pst* DC3000 AvrRps4 (13 h after Dex treatment), as indicated. Western blot shows EDS1 protein levels in total and nuclei-enriched fractions in wt (Ler) and the indicated transgenic lines expressing EDS1-YFP-GR fusion protein. PonceauS staining of the membrane shows equal loading. PEPC and HistoneH3 were used respectively as cytosolic and nuclear markers. The nuclei-depleted fraction from wt (Ler) untreated plants was loaded together with nuclei-enriched fractions to detect potential cytosolic contamination by anti-PEPC signal. **(B)** *EDS1* transcript accumulation in EDS1-YFP-GR transgenic lines 8 h after triggering RPS4 resistance. Four-week-old plants untreated (grey bars) or pretreated with Dex (5 h, black bars) were spray-inoculated with *Pst* DC3000 AvrRps4. Leaf samples were collected from untreated plants at 0 h (T0) or 8 h after pathogen inoculation. Bars represent means and standard deviations of two or three biological replicates. Expression was normalized against the endogenous control gene *UBIQUITIN*. **(C)** Western blot showing YFP-GR fusion protein levels in untreated and Dex treated (13 h) plants. **(D)** Western blot showing EDS1 total protein levels in wt (Ler) plants untreated, 13 h after Dex treatment and 8 h post inoculation with *Pst* DC3000 AvrRps4, as indicated. Ponceau S staining of membrane shows equal loading. Molecular weights of protein markers are shown on the right of panels A, C and D.
(1.06 MB TIF)

Table S1 Total number of transcripts altered by avirulent Pst DC3000 AvrRps4 in wild type and their dependence on EDS1. This table shows the total number of transcripts altered by >2,5 fold at 6 h after inoculation with Pst DC3000 AvrRps4 (compared to mock treatment) in wild type followed by the number that are dependent on EDS1 (>2,5 fold less induced or repressed in eds1-1/mock compared to WT/mock) (http://www.ebi.ac.uk/arrayexpress/). Data were extracted from Bartsch et al. [5].
(0.03 MB DOC)

Table S2 Genes transcriptionally induced or repressed in an EDS1-dependent manner. Data were extracted from Bartsch et al. [5]. (http://www.ebi.ac.uk/arrayexpress/)
(0.03 MB DOC)
