# Supplementary figures and images for: Balanced Nuclear and Cytoplasmic Activities of EDS1 Are Required for a Complete Plant Innate Immune Response
Source: PLoS Pathog. 2010 Jul 1;6(7):e1000970. doi: 10.1371/journal.ppat.1000970 (PMC2895645; doi:10.1371/journal.ppat.1000970)

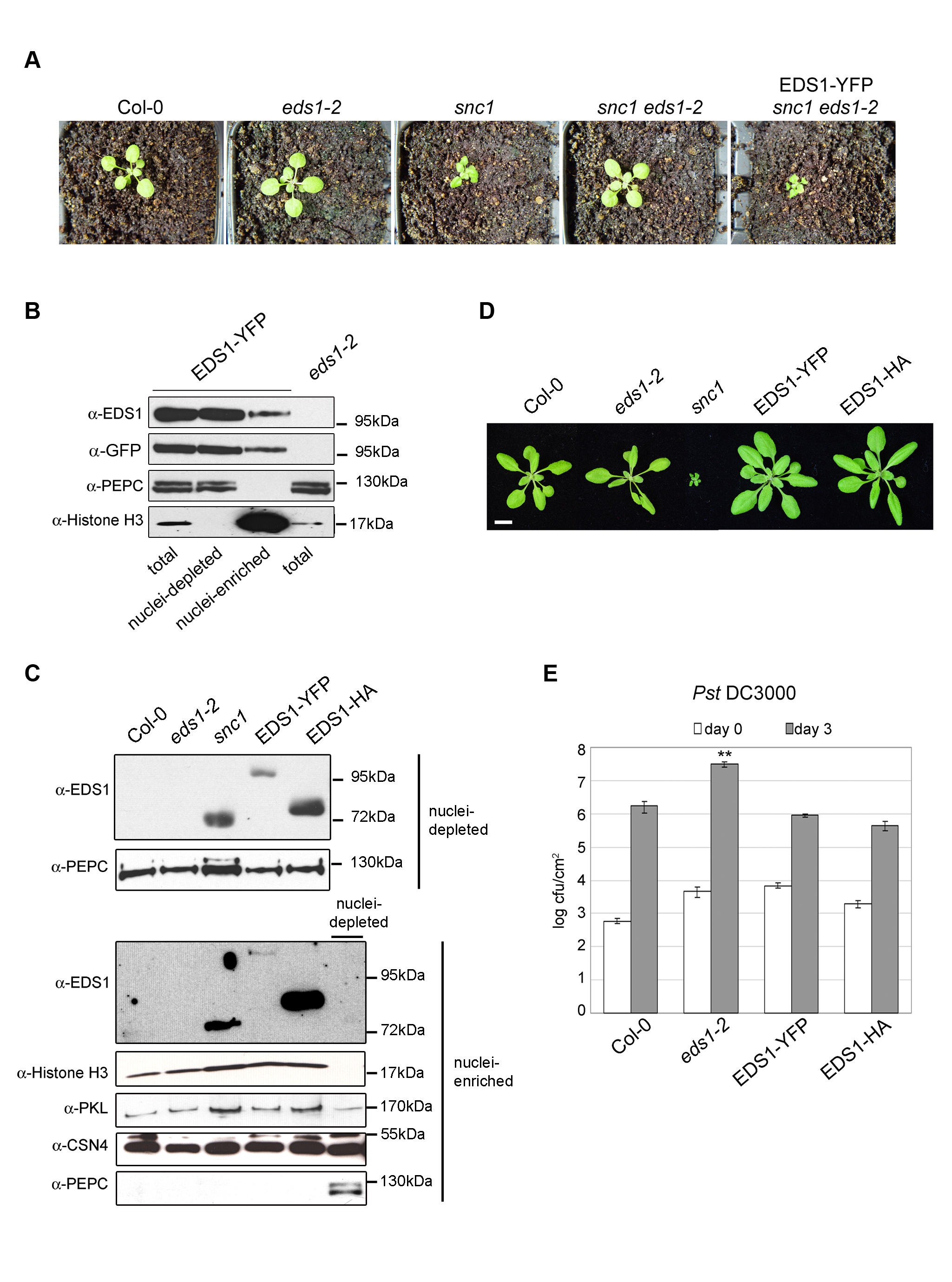

Supplement: Figure S1 — Increased EDS1 protein levels do not lead to constitutive resistance. (A) Picture of 3-week-old soil grown plants showing that Col eds1-2 suppresses snc1 phenotypes and expression of EDS1-YFP (driven by the EDS1 native promoter) in snc1 restores them. (B) Western blot showing levels of EDS1-YFP fusion protein in total, nuclei-depleted and nuclei-enriched fractions prepared from 4-week-old soil grown plants. Nuclei-enriched fractions are 30× concentrated (v/v) compared to nuclei-depleted fractions. Samples were loaded twice on the same gel and blotted together. One half of the membrane was probed with anti-EDS1, the other with anti-GFP antibodies. PEPC and Histone H3 were used respectively as cytosolic and nuclear markers. Molecular weights of protein markers are shown on the right. (C) Western blot showing EDS1 protein levels in nuclei-depleted and nuclei-enriched fractions prepared from healthy tissue of the indicated genotypes over expressing EDS1. PEPC was used as cytosolic marker. Chromatin associated Histone H3, the chromatin remodeler protein PKL (PICKLE) and the CSN4 subunit of COP9 signalosome (chromatin non-associated protein) were used as nuclear markers. The nuclei-depleted fraction from wt (Col-0) untreated plants was loaded together with nuclei-enriched fractions in order to monitor potential cytosolic contamination by anti-PEPC signal. Molecular weights of protein markers are shown on the right. (D) Picture of 4-week-old soil grown plants of the indicated genotypes showing normal growth of all lines except snc1. (E) Bacterial infection assay. 4-week-old soil grown plants were spray-inoculated with Pst DC3000 AvrRps4 and bacterial titers determined 0 and 3 d post inoculation. Bars represent means of 3 replicates ± standard error. ** p value<0.001. (3.04 MB TIF) [file ppat.1000970.s001.tif]

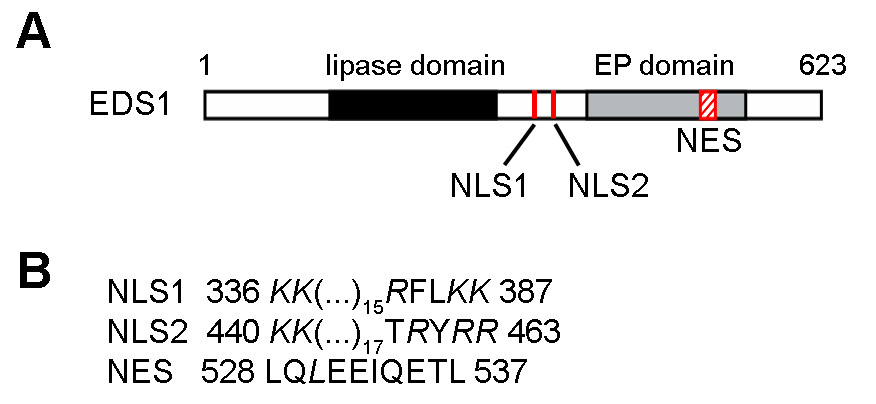

Supplement: Figure S2 — Domain structure of EDS1 protein depicting putative localization signals. (A) Black box represents conserved lipase-like domain, red lines show positions of predicted nuclear localization signals (NLSs) and dashed red box represents predicted nuclear export signal (NES). (B) Amino acid sequences of EDS1 putative NLS and NES motifs. Residues in italics were mutagenized to test the functionality of the signal. Lysine (K) and arginine (R) residues in NLS1 and NLS2 were replaced with glutamine and Leucine (L) 530 in NES sequence was replaced with alanine. (0.03 MB TIF) [file ppat.1000970.s002.tif]

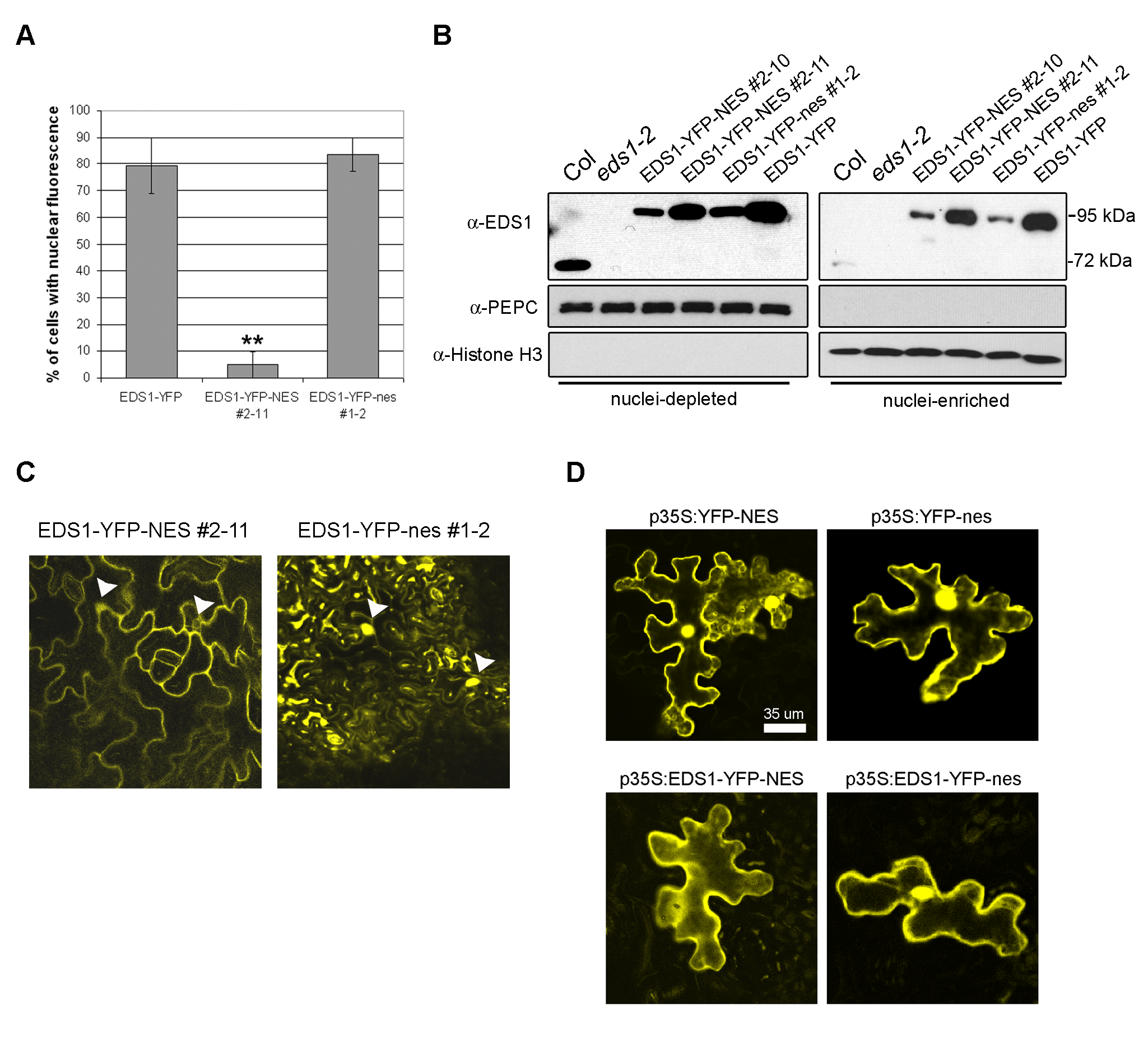

Supplement: Figure S3 — Nucleo-cytoplasmic partitioning of the EDS1-YFP-NES and EDS1-YFP-nes fusion proteins. (A) Percentage of cells showing detectable nuclear fluorescence in the indicated stable transgenic lines from at least 100 epidermal cells in three individual plants per genotype. (B) Western blot showing EDS1 protein levels in nuclei-depleted and nuclei-enriched fractions. PEPC and Histone H3 were used respectively as cytosolic and nuclear markers. Molecular weights of protein markers are shown on the right. (C) Confocal images showing the subcellular distribution of the EDS1-YFP-NES and EDS1-YFP-nes fusion proteins in leaves of healthy transgenic plants. Bar is 15 µm. Accumulation of EDS1-YFP-NES fusion protein in and around nuclei can be observed and is depicted with a white arrowhead. (D) Confocal images showing the subcellular distribution of the indicated fusion proteins expressed transiently in Col eds1-2 leaf epidermal cells by particle bombardment. (1.50 MB TIF) [file ppat.1000970.s003.tif]

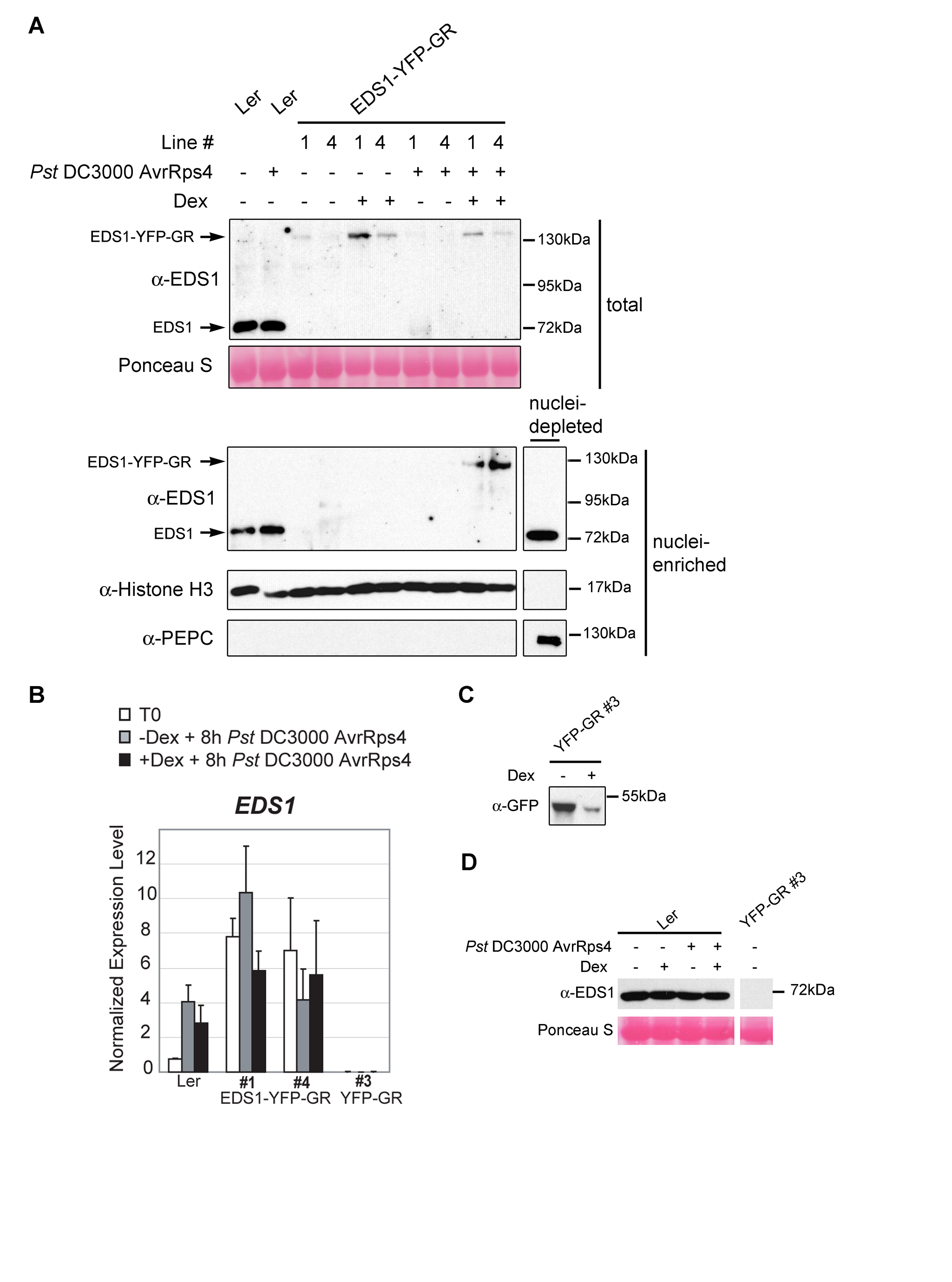

Supplement: Figure S4 — Accumulation of EDS1 and EDS1-YFP-GR fusion after Dex treatment. (A) Identical samples as used in Figure 6A probed with anti-EDS1. Four-week-old plants were pretreated with Dex (5 h) and spray-inoculated with Pst DC3000 AvrRps4. Protein samples were prepared 8 h post inoculation with Pst DC3000 AvrRps4 (13 h after Dex treatment), as indicated. Western blot shows EDS1 protein levels in total and nuclei-enriched fractions in wt (Ler) and the indicated transgenic lines expressing EDS1-YFP-GR fusion protein. PonceauS staining of the membrane shows equal loading. PEPC and HistoneH3 were used respectively as cytosolic and nuclear markers. The nuclei-depleted fraction from wt (Ler) untreated plants was loaded together with nuclei-enriched fractions to detect potential cytosolic contamination by anti-PEPC signal. (B) EDS1 transcript accumulation in EDS1-YFP-GR transgenic lines 8 h after triggering RPS4 resistance. Four-week-old plants untreated (grey bars) or pretreated with Dex (5 h, black bars) were spray-inoculated with Pst DC3000 AvrRps4. Leaf samples were collected from untreated plants at 0 h (T0) or 8 h after pathogen inoculation. Bars represent means and standard deviations of two or three biological replicates. Expression was normalized against the endogenous control gene UBIQUITIN. (C) Western blot showing YFP-GR fusion protein levels in untreated and Dex treated (13 h) plants. (D) Western blot showing EDS1 total protein levels in wt (Ler) plants untreated, 13 h after Dex treatment and 8 h post inoculation with Pst DC3000 AvrRps4, as indicated. Ponceau S staining of membrane shows equal loading. Molecular weights of protein markers are shown on the right of panels A, C and D. (1.06 MB TIF) [file ppat.1000970.s004.tif]
