# Supplementary material for: Balanced Nuclear and Cytoplasmic Activities of EDS1 Are Required for a Complete Plant Innate Immune Response
Source: PLoS Pathog. 2010 Jul 1;6(7):e1000970. doi: 10.1371/journal.ppat.1000970 (PMC2895645; doi:10.1371/journal.ppat.1000970)
Supplement: Table S1 — Total number of transcripts altered by avirulent Pst DC3000 AvrRps4 in wild type and their dependence on EDS1. This table shows the total number of transcripts altered by >2,5 fold at 6 h after inoculation with Pst DC3000 AvrRps4 (compared to mock treatment) in wild type followed by the number that are dependent on EDS1 (>2,5 fold less induced or repressed in eds1-1/mock compared to WT/mock) (http://www.ebi.ac.uk/arrayexpress/). Data were extracted from Bartsch et al. [5]. (0.03 MB DOC) [file ppat.1000970.s005.doc]

**Table S1. Total number of transcripts altered by avirulent Pst DC3000 AvrRps4 in wild type and their dependence on EDS1**

|  | Number of genes in wild-type | Number of genes that are EDS1-dependent |
| --- | --- | --- |
| induced | 924 | 721 (78%) |
| repressed | 1694 | 1224 (72%) |

This table shows the total number of transcripts altered by >2,5 fold at 6 h after inoculation with *Pst* DC3000 AvrRps4 (compared to mock treatment) in wild type followed by the number that are dependent on EDS1 (>2,5 fold less induced or repressed in eds1-1/mock compared to WT/mock) (http://www.ebi.ac.uk/arrayexpress/). Data were extracted from Bartsch et al. [5]
