# Supplementary material for: Balanced Nuclear and Cytoplasmic Activities of EDS1 Are Required for a Complete Plant Innate Immune Response
Source: PLoS Pathog. 2010 Jul 1;6(7):e1000970. doi: 10.1371/journal.ppat.1000970 (PMC2895645; doi:10.1371/journal.ppat.1000970)
Supplement: Table S2 — Genes transcriptionally induced or repressed in an EDS1-dependent manner. Data were extracted from Bartsch et al. [5]. (http://www.ebi.ac.uk/arrayexpress/) (0.03 MB DOC) [file ppat.1000970.s006.doc]

**Table S2. Genes transcriptionally induced or repressed in an EDS1-dependent manner**

**A. Selected defense-marker genes transcriptionally induced by avirulent *Pst*** DC3000 AvrRps4 in an EDS1-dependent manner

| AGI Code | Fold Change in WT | Fold Change in *eds1-1* | Description |
| --- | --- | --- | --- |
| At3g52430 | +60,8 | -1,54 | PAD4 (phytoalexin deficient4); lipase/ protein binding / triacylglycerol lipase |
| At1g74710 | +29,7 | +1,11 | ICS1 (isochorismate synthase1); isochorismate mutase |
| At5g26920 | +99,8 | +1,89 | CBP60G (CaM-binding protein 60-like.g); calmodulin binding |
| At2g14610 | +4,42 | -1,24 | PR1 (pathogenesis-related1) |
| At3g48090 | +15,9 | +1,31 | EDS1 (enhanced disease susceptibility1); lipase/ signal transducer/ triacylglycerol lipase |
| At1g19250 | +152 | -9,03 | FMO1 (flavin-dependent monooxygenase1); FAD binding / NADP or NADPH binding / electron carrier/ flavin-containing monooxygenase/ monooxygenase/ oxidoreductase |
| At5g13320 | +394 | -2,18 | PBS3 (avrPphB susceptible3) |

**B. Selected defense-marker genes transcriptionally repressed by avirulent *Pst*** DC3000 AvrRps4 in an EDS1-dependent manner

| AGI Code | Fold Change in WT | Fold Change in *eds1-1* | Description |
| --- | --- | --- | --- |
| At2g26330 | -43,3 | -1,11 | ER (erecta); transmembrane receptor protein kinase |
| At3g46130 | -5,19 | +2,58 | MYB48 |
| At5g15410 | -7,19 | -1,19 | DND1 (defense no death1); calcium channel/ calmodulin binding / cation channel/ cyclic nucleotide binding / intracellular cAMP activated cation channel/ intracellular cyclic nucleotide activated cation channel/ inward rectifier potassium channel |
| At1g66140 | -14,0 | -1,71 | ZFP4 (zinc finger protein4); nucleic acid binding / transcription factor/ zinc ion binding |

Data were extracted from Bartsch et al. [5]. (http://www.ebi.ac.uk/arrayexpress/)
